# Migration dynamics of acoustic-tagged hatchery and natural atlantic salmon smolts

**DOI:** 10.1186/s40850-026-00278-y

**Published:** 2026-07-23

**Authors:** James P. Hawkes, Graham S. Goulette, Alejandro Molina-Moctezuma

**Affiliations:** 1 NOAA’s National Marine Fisheries Service, 5 Godfrey Drive, Suite #2, Orono, Maine, USA; 2 University of Tennessee, School of Natural Resources, 2431 Joe Johnson Drive, 474 Agriculture and Natural Resources Building, Knoxville, TN USA

**Keywords:** Atlantic salmon, Smolt migration, Survival, Acoustic telemetry, Rearing origin, Penobscot river, River discharge

## Abstract

We investigated the migration dynamics of two Atlantic salmon (*Salmo salar*) smolt origin groups (hatchery, *n* = 374; natural, *n* = 231) in the Penobscot River Estuary and Bay, Maine, U.S.A., during the springs of 2010–2012. Smolts were acoustic-tagged and tracked for approximately 100 km to evaluate differences in movement rate, migration duration, path choice, and survival. While the behavioral differences between origin groups were not consistent, differences in survival were evident. Using a Cormack-Jolly-Seber mark-recapture model, we found that rearing origin and river discharge were the primary factors influencing smolt survival. Survival decreased with increasing river discharge across the observed range. Hatchery smolts (cumulative survival: 0.40–0.69) presented higher survival rates than their natural counterparts did (0.32–0.64), a finding that is inconsistent with much of the literature on hatchery versus river-reared fish. We hypothesize that the collection method for natural smolts at a dam bypass facility, which involves novel handling and dam interaction, may have contributed to their lower survival, potentially masking any inherent advantage over hatchery smolts. These findings underscore the importance of minimizing dam-related stressors and informing management actions aimed at improving the survival of outgoing smolt cohorts.

**Supplementary Information:**

The online version contains supplementary material available at https://doi.org/10.1186/s40850-026-00278-y.

## Impact statement

Improved dam passage is critical for increasing overall restoration efforts.

**Supplementary Information:**

The online version contains supplementary material available at https://doi.org/10.1186/s40850-026-00278-y.

## Background

Populations of migratory freshwater fish worldwide have declined by an average of 76% over the past 50 years, a decline attributed to human impacts (i.e., migration barriers, climate, and overharvest; [12]). Numerous attempts have been made to reverse species declines, including species protection, habitat restoration and population supplementation. Salmonid restoration efforts have been at the forefront for decades; objectives range from creating self-sustaining or harvestable populations, with many efforts focused on supplementing wild populations. These hatchery supplementation efforts experience different levels of success and efficacy, and their utility is often questioned [2, 3, 41]. Despite ongoing discussions regarding the perceived inferiority of hatchery-origin Atlantic salmon, stocking practices remain a common and important conservation tool [63].

For example, Atlantic salmon in the United States, at the southern extent of their range, are at critically low abundance, and the Gulf of Maine Distinct Population Segment (GOM DPS) is afforded protection under the U.S. Endangered Species Act [68]. Populations to the south are considered extirpated [27, 67]. Hatchery supplementation supports remnant populations in Maine because natural production has become limited. Without supplementation, the remaining U.S. populations would also become extirpated [40, 48, 49]. Despite hatchery intervention, adult returns to Maine remain static, with 1,000 to 1,500 adult Atlantic salmon returning annually [67].

Among the seven river-specific stocks maintained by the U.S. Fish and Wildlife Service (USFWS), the greatest number of U.S.-origin adult Atlantic salmon return annually to the Penobscot River. The USFWS releases approximately 500,000 age-1 hatchery smolts into the Penobscot River. The total annual return average is less than 1,200 and the smolt-to-adult return rate for multiple sea-winter adults has been less than 0.5% over the past two decades [67]. Natural smolts (products of wild spawning (limited), egg planting, or stocked fry) contribute to the migrating cohort each spring, so natural smolts largely originate from the hatchery, but experience river rearing. The proportion of natural migrants within the Penobscot River is not clearly understood because of the difficulty of performing quantitative assessments [16]. While data regarding age structure in the Penobscot River is limited, sampling of emigrating smolts (2001–2005) determined approximately 80% of existing smolts were Age 2 with the remaining majority Age 3 [16], which is consistent with other GOM DPS populations [34, 66]. Migration windows of smolts within the Penobscot River and GOM DPS are consistently found within the timeframe of mid-April though early-June, with some annual variability observed, but the peak is often found around the first or second week in May [15, 34, 66]. Nearly all natural Atlantic salmon smolts within the drainage must successfully pass at least one, and potentially up to eight (at the time of this study), dams prior to estuary entry. Interactions with dams can result in both immediate and latent mortality [24, 46, 57–60]. This highlights the challenge in evaluating the efficacy of different restoration strategies and underscores the need for direct comparisons of natural and hatchery smolt performance.

Adult returns ultimately define Atlantic salmon smolt stocking as a measure of success [29]. Telemetry, however, provides an early indication of cohort success in the early marine environment [36–38, 61, 65]. While telemetry assessments of hatchery smolts are common in the Penobscot River [17, 53], evaluations of natural smolts have been minimal due to collection difficulties during migration. Stich et al. [59] conducted the only assessment of hatchery and wild origins in the Penobscot, evaluating smolt behavior and survival during estuary migration but only after passing several dams. To address this critical need and specifically minimize the influence of dam passage on smolt performance, this study aimed to describe and compare the migration rate, duration, path choice, and survival of hatchery and natural smolts within the Penobscot River watershed.

## Methods

### Study area

**Fig. 1:**
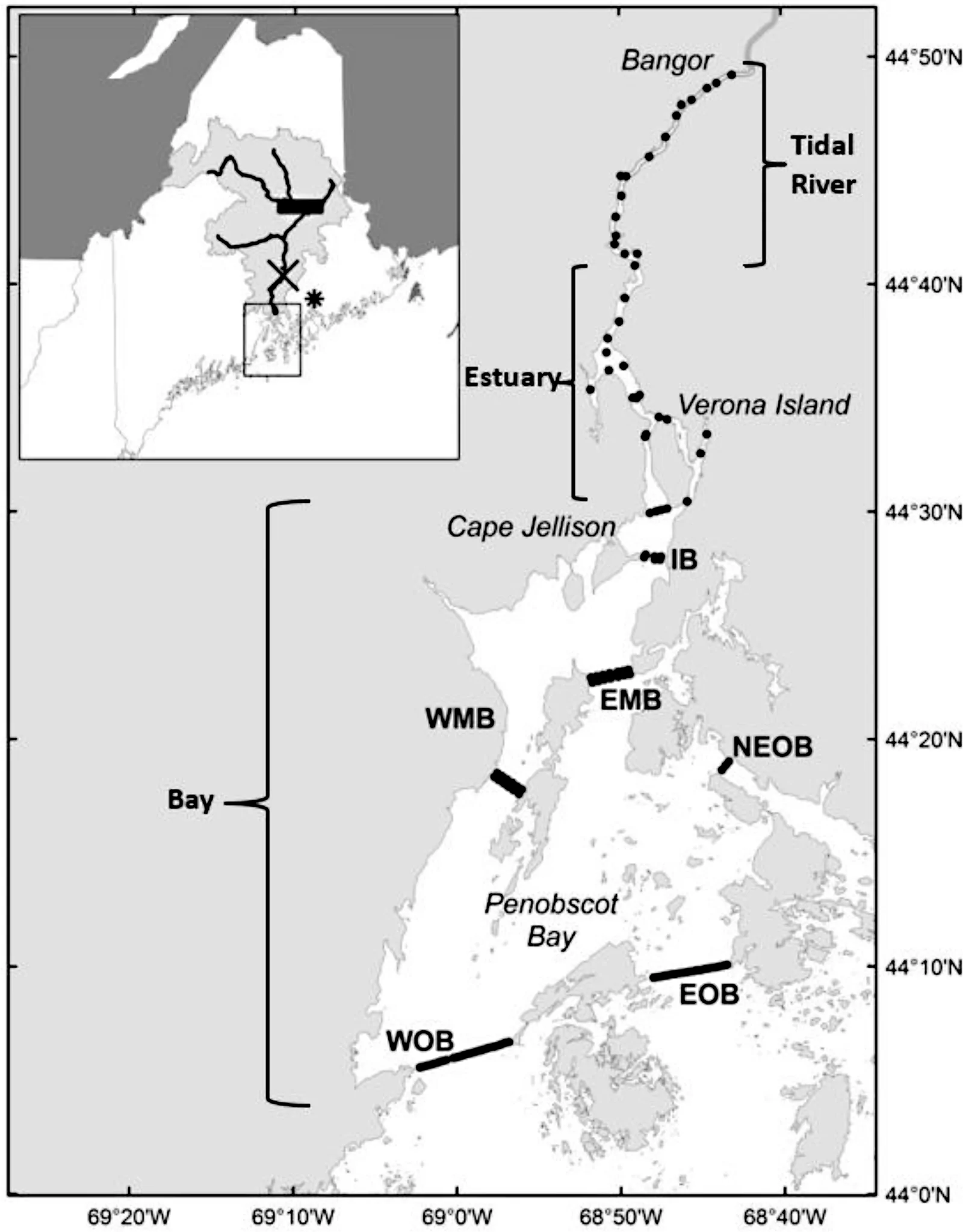
Study area within the Penobscot River, Maine. Plotted telemetry network represents coverage for each year of study from 2010–2012. The study area is delineated into five reaches: Tidal River (Tidal RIV), Estuary (EST), Inner Bay (IB), Middle Bay (which includes Western Middle Bay (WMB), Eastern Middle Bay (EMB)), and Outer Bay (which includes Western Outer Bay (WOB), Eastern Outer Bay (EOB), and Northeastern Outer Bay (NEOB)). Black dots are receiver locations. Insert – “▀” is dam site (bypass trapping facility) where natural smolts were collected, “ * ” represents U.S. Fish and Wildlife Green Lake National Fish Hatchery, Ellsworth, where hatchery smolts were reared and collected, and “ X ” represents release location of tagged fish

The Penobscot watershed covers approximately 22,000 km^2^ and is the largest drainage area within Maine, USA (Fig. 1). The mainstem river drops 70 m in elevation from the confluence of the East and West branches to the head-of-tide, which spans a distance of 120 km. The estuary is 51 km long from the head-of-tide to where it empties into Penobscot Bay [4]. The Penobscot estuary experiences significant mixing that varies with freshwater discharge and tide height, which results in temperature and salinity shifts [19]. Penobscot Bay contains two main channels (east and west) bisected by a series of islands. The spring freshet is the primary driver for subtitle circulation with discharge and wind found to be drivers of surface outflows in the eastern and western parts of the bay [73]. During the study period (April 30 to May 30), the median annual discharge was 227.7 m³/s in 2010, 1,112.9 m³/s in 2011, and 512.5 m³/s in 2012 the median timeseries value for this site (1970–2009) is 494.1 m³/s [69].

### Acoustic receiver array

Each year, we deployed up to 129 VR receivers (VR2 and VR2W; Innovasea Bedford, Nova Scotia Canada) throughout the Penobscot River, Estuary, and Bay (Fig. 1). We deployed receivers individually (a node) or in a continuous series with overlapping coverage (a curtain), as described in Kocik et al. [33].

The estuary mouth at river kilometer (rkm) 0.0 (Fig. 1) has western (main channel) and eastern routes, which are separated by Verona Island and transition into the inner bay (IB) at Cape Jellison. Further seaward, the middle bay channels are partitioned into the western middle bay (WMB) and eastern middle bay (EMB), collectively identified as the “middle bay array”. The outer bay has three possible exit points for emigrating postsmolts (the period just after smolts have left the river, entering areas of increased salinities; [42]): the western outer bay (WOB), eastern outer bay (EOB) and northeastern outer bay (NEOB), which are collectively identified as the “outer bay array” (Fig. 1). The receivers within each array were spaced an average of 400 m (ranging from 200 to 600 m), providing coverage for high detection efficiency, which is documented to range from 95 to 99% [18].

### Collection, tagging, and release of Atlantic salmon smolts

Paired releases of hatchery and natural smolts occurred each of the three years (2010–2012). We collected natural smolts at the downstream bypass at the first mainstem dam encountered during emigration, Weldon dam, at the Mattaceunk Hydroelectric Project (FERC No. 2520; Lat 45.47°, Lon − 68.41°; rkm 149). During the three years of this study, 11.5% (30/261) of the natural smolts collected at the bypass trap were excluded because they were dead or moribund; we selected only those without visible injury or irregular behavior for surgery, thus ensuring that the survival estimates represented a minimum impact scenario. Smolts migrating downstream were trapped, collected each morning, and placed into an insulated Xactics tank (Cornwall, Ontario) for transport to the surgical location where they were tagged and released on the day of capture.

We collected hatchery smolts from the USFWS Green Lake National Fish Hatchery, Ellsworth, Maine. These smolts are part of an age-1 accelerated growth program that produces smolts in 18 months, resulting in fish that are longer and heavier than the older natural smolts in the river [16]. Smolts are raised outdoors under a canopy in 6 m circular cement pools using water drawn from Green Lake (Lat 44.64°, Lon − 68.51°). Smolts were collected each morning and placed into an insulated Xactics TM tank (Cornwall, Ontario) for transport to the surgical location. Smolts were surgically implanted with V9–6 L transmitters (2.9 g in air, 21 mm in length, 9 mm in diameter; Amirix Systems Inc.) over a three-year period. Prior to surgery, Atlantic salmon smolts were anesthetized using tricaine methanesulfonate (MS-222; 100 mg/L) buffered with sodium bicarbonate (NaHCO_3;_ 1:1 concentration to MS-222) to neutral pH, until loss of equilibrium was achieved (~ 2–3 min). Surgical procedures followed the methodologies described by Kocik et al. [33]. The transmitters emitted coded acoustic pulses, initially every 20 to 40 s (30-second average) for 35 days, then every 60 to 80 s (70-second average) to extend the battery life.

We measured smolts (FL; 1 mm), weighed them (wet weight; 0.1 g), and assigned them a “smolt score” (physiological proxy) on the basis of observed characteristics specific to physiological readiness (e.g., Gill NKA activity; [42]). We assigned scores ranging from 1 to 6, with 1 indicating no silvering and heavy parr marks (presmolt) and 6 indicating loss of silvering and deciduous scales (desmoltifying; [42] and NOAA’s SOP, unpublished). Post-surgery, we held smolts for an average of three hours, prior to release, with no concerns of tags being expelled (though previous investigations and literature) within the timeframe after surgery through exiting the array [7, 23, 36], We released smolts at a local boat launch in Brewer, ME (rkm 44; Lat 44.82° Lon − 68.72°; Fig. 1). We performed all surgeries between April 30 and May 27.

To limit transmitter burden, we selected smolts using the criterion of transmitter weight not exceeding 8% of smolt weight established in Lacroix et al. [36]. We calculated the percent tag burden via Eq. 1, where SW = smolt weight (g) and 2.9 represents the transmitter weight in air (g).

1 $$\:\%\:Tag\:burden\:=\:(2.9\:g/\:SW)\:\times\:\:100$$

### Environmental data collection and sources

We obtained instream river temperatures from the USGS National Water Information System website (gage #1036390) [70]. We obtained estuary temperatures from Onset Hobo water temperature data loggers (instrument accuracy, ± 0.2 °C; [19] Bourne, Massachusetts) deployed at rkm 10.0 and rkm − 3.9 at a depth of 1 m. For the outer array, we obtained temperature data from the University of Maine Ocean Observing System buoy F01 (mouth of Penobscot Bay at a depth of 2 m; [64]).

We obtained discharge data from the USGS National Water Information System website (gage #1034500) [69].

## Analysis

### Temperature and smolt detection data merge

We determined the thermal experience (°C) for smolts through the network in the tidal river, estuary, inner bay and outer bay by merging telemetry detection (date/time) and temperature (date/time) data, with no interpolation of temperature when fish went undetected. Comparisons of temperatures across environments were made, as were comparisons of differences from one environment to the next. We compared these data via nonparametric Kruskal‒Wallis tests (α = 0.05) because the data were not normally distributed.

### Discharge

Discharge (m³/s) data was summarized using a line graph for each year, as well as incorporated into model development. These data are not presented for individual movements, but to provide context for conditions experienced by migrating salmon.

### Fish data

#### Migration movement rate and duration

To mitigate impacts from handling and surgical procedures, data summaries only included smolts detected 5.2 km downstream of release. Only postsmolts detected on the outer bay array (Fig. 1) were considered successful and included in this analysis.

The migration rates of smolts/postsmolts through the receiver network were calculated in kilometers per hour (km/h). At defined receiver locations, we subtracted the first detection data (date/time) at a downstream site from the last detection data (date/time) at an upstream site. We calculated the migration rate via Eq. 2, where distance in kilometers (km) was divided by the time in hours (h) to calculate the movement rate:

2 $$\:Migration\:Movement\:Rate\:(km/h)\:=\:Distance/h$$

We calculated the duration (in days = d) of smolt travel through the network via Eq. 3, where the date/time at first detection outer array (FDO) was subtracted from the date/time from the starting point (SP):

3 $$\:Duration\:\left(d\right)\hspace{0.17em}=\hspace{0.17em}FDO\:-\:SP$$

We compared the migration rate and duration data via the nonparametric Kruskal‒Wallis test (α = 0.05) because the data were not normally distributed.

### Path choice and distribution

Path choice was defined by detection through the receiver network (Fig. 1). To test for differences in detections across receiver sites in the outer arrays, we used a generalized linear model with a Poisson distribution (GLM): log(counts) ~ β_0_ + β_1_ × site within array. We did not summarize the path choice of postsmolts around Verona Island due to receiver loss (2011) and minimal use by postsmolts on the eastern channel during other years (natural: *n* = 4 (2010), 3 (2012); hatchery: *n* = 3 (2010), 6 (2012)).

### Survival estimates

Individual detection histories for smolts and postsmolts were used to estimate apparent survival (hereafter, survival) from mark–recapture models via a Cormack–Jolly–Seber approach, implemented in the MARK program [72] via the RMark package [35] in R (version 4.5.3; [52]). We estimated survival through two tidal riverine, four estuarine, and three bay reaches. Each reach consisted of multiple aggregated receivers in order to maximize detection probabilities. As smolts move unidirectionally (no reversals are observed between defined reaches) during their migration, ceasing downstream movement can be identified as mortality; therefore, we used receiver stations (Fig. 1) as capture occasions and estimated survival in the space intervals between the receivers, i.e., a reach (see [24, 45, 58]). To account for the varying lengths of each reach, the reach interval length (rkm) was explicitly incorporated into the models. This allowed us to compare survival per rkm (Φ_rkm_) in different reaches. To obtain survival for the whole reach, the rkm survival was raised to the power of the reach length (Φ_reach_ = Φ_rkm_^length(rkm)^), and to obtain the error structured for the whole reach, the delta method was used. We estimated the overdispersion parameter (ĉ) via the median method in program MARK to assess the goodness of fit of the survival models [13]. Because ĉ was lower than 1.5, we used an information-theoretic approach to model selection based on Akaike’s information criterion corrected for sample size (AICc).

Survival models comprised a suite of a priori selected covariates representing temporal, environmental, and smolt-level characteristics across the three study years. We used a two-phase approach to model selection and inference.

In the first phase, survival (Φ) was held constant across all candidate models, whereas the detection probability (p) was allowed to vary. The candidate models for p included reach, year, and origin. Individual-level covariates were excluded from the detection probability models on the assumption that the characteristics of individual fish should not meaningfully influence the probability of being detected in an array. All additive and interactive combinations of the three variables were evaluated and compared using AIC. The best-supported detection probability model from this phase was then carried forward and held constant in all subsequent models.

In the second phase, the detection probability (p) was fixed to the best model identified in phase one, and candidate models were built to evaluate the relative importance of different covariates on survival (Φ). All the models included reach as a variable to account for differences in the interval distance between receiver stations. We also constructed a null model in which reach was the only predictor. The remaining models incorporated group-level covariates, individual-level covariates, environmental covariates, or combinations thereof. The group-level variables included origin (hatchery vs. natural) and year (2010, 2011, 2012). Individual-level covariates included the smolt score and tag burden. The environmental covariates included discharge and temperature differential. Previous exploratory models included Fork-Length as an individual covariate, which was confounded with origin, and thus were removed.

To avoid overparameterization given the number of detection events relative to the model parameters, we constrained the candidate models to include no more than two covariates (beyond reach) in any single model. This constraint ensured that the models remained parsimonious and that the parameter estimates were reliable. We ran a total of 30 candidate models, including the null model, single-covariate models, and all combinations of up to two covariates. For discharge specifically, we also tested a quadratic term ($\:{\beta\:}_{1discharge}+\:{\beta\:}_{2discharg{e}^{2}}$) to account for nonlinear effects on survival that have been documented in migrating smolts [11, 46, 59]. Model selection was based on AICc, ΔAICc, and the relative likelihood of each model (Akaike weight w_i_; [8]). Using the best model from the second phase of model selection, we estimated Φrkm for each reach (r) of the study area during each year. We estimated cumulative survival with the following equation:$\:Cumm\left({\varPhi\:rkm}_{r}\right)={\prod\:}_{1}^{r}{{\varPhi\:rkm}_{r}}^{{length\left(rkm\right)}_{r}}$

Finally, we explored the mortality per environment (tidal, estuary, and bay). Under normal circumstances, survival cannot be estimated for the last interval in CJS models owing to confounding effects of survival and recapture. Therefore, we deployed three sentinel (V9-6 L range test) transmitters at three distances from the receivers to independently test the detection probabilities of the outer array. We observed that this array had a probability of detection of 0.99 (J.P. Hawkes and G.S. Goulette, National Marine Fisheries Service, unpublished); therefore, we fixed p for the last reach (0.99) and estimated Φrkm for the last reach.

To formally evaluate whether apparent survival differed among origins, we conducted a nested model comparison using Program MARK through RMark. We compared the selected Cormack-Jolly-Seber model that included origin as a predictor of apparent survival with an otherwise identical reduced model in which origin was removed from the survival component. The detection probability structure was held constant between the two models. We evaluated the contribution of origin using a likelihood ratio test, calculated as the difference in model deviance between the reduced and full models, with degrees of freedom equal to the difference in the number of estimated parameters.

### Comparative assessment

To compare our results with those of other published studies, we standardized survival to habitat-specific results for freshwater (tidal rivers), estuaries and bays. These data were summarized via median per-rkm values within environments, and these data were compared with Fig. 1 in Thorstad et al. [62].

## Results

### Smolt characteristics

Surgical procedures resulted in no immediate mortality. The Hatchery (*n* = 374) smolts presented a median FL of 195.0 mm (95% CI = 187–204 mm) and a mass of 74.4 g (95% CI = 66.0–84.1 g). The natural smolts (*n* = 231) had a median FL of 172.0 mm (95% CI = 161–184 mm) and a mass of 47.4 g (95% CI = 41.2–59.4 g). We found significant differences in FL and mass between the groups each year (*P* < 0.01; Table 1). We also found significant differences in FL and mass across years for hatchery smolts (*P* < 0.001), whereas for natural smolts, there were no differences in FL between 2010 and 2011 (*P* = 0.93), but differences were observed in 2012 versus the other two years (*P* < 0.004), and no differences in mass (*P* < 0.002) were detected. The percent transmitter burden also differed significantly by origin group annually (*P* < 0.001; Table 1), with hatchery smolts averaging 3.9% (95% CI = 3.5–4.4%) and natural smolts averaging 6.1% (95% CI = 4.9–7.0%). The smolt scores remained consistent across years within origin groups; most hatchery smolts scored 5 (85%), whereas approximately half (51%) of the natural smolts scored 4 (Table 2).

**Table 1:** Biological Statistics of telemetry tagged smolts

Year	Origin	*n*	Release Dates	Median Fork Length (mm)	C.I. Fork Length (mm)	KW Test (Test Stat/*P* Value)	Median Mass (g)	C.I. Mass (g)	KW Test (Test Stat/*P* Value)	Median % Burden (Air)	C.I. % Burden (Air)	KW Test (Test Stat/*P* Value)
2010	HTC	99	May 5 - May 8	192	186–202	56.0/ *P* < 0.001	73.2	67.2–84.9	79.0/ *P* < 0.001	4.0	3.4–4.3	79.0/ *P* < 0.001
2010	NR	100		175	164–186		49.4	41.6–61.6		5.9	4.7–7.0	
2011	HTC	154	May 16 - May 27	194	187–201	41.0/ *P* < 0.001	69.3	67.1–77.1	47.2/ *P* < 0.001	4.2	3.8–4.7	38.8/ *P* < 0.001
2011	NR	50		175	160–189		52.9	39.5–63.2		5.5	4.6–7.4	
2012	HTC	121	Apr 30 - May 3	200	190–207	123.8 / *P* < 0.001	80.3	72.1–92.4	124.89/ *P* < 0.001	3.6	3.1–4.0	124.9/ *P* < 0.001
2012	NR	81		166	160–176		44.7	41.2–53.7		6.5	5.4–7.0	
Total	HTC	374	2010–2012	195	187–204	229.9/ *P* < 0.001	74.4	66.0–84.1	248.1/ *P* < 0.001	3.9	3.5–4.4	248.2/ *P* < 0.001
	HTC	231		172	161–184		47.4	41.2–59.4		6.1	4.9–7.0	

HTC = Hatchery; NR = Natural

**Table 2:** Smolt score of telemetry tagged smolts by year

	Smolt Score					
HTC	1	2	3	4	5	6
2010	0.0%	0.0%	0.0%	6.1%	91.9%	2.0%
2011	0.0%	0.0%	0.0%	11.0%	89.0%	0.0%
2012	0.0%	0.0%	2.5%	23.1%	74.4%	0.0%
Mean	0.0%	0.0%	0.8%	13.4%	85.1%	0.7%
**NR**						
2010	0.0%	0.0%	2.0%	55.0%	43.0%	0.0%
2011	0.0%	0.0%	12.2%	46.9%	40.8%	0.0%
2012	0.0%	0.0%	38.1%	51.2%	10.7%	0.0%
Mean	0.0%	0.0%	17.4%	51.0%	31.5%	0.0%

HTC = Hatchery; NR = Natural

### Temperature and discharge

**Fig. 2:**
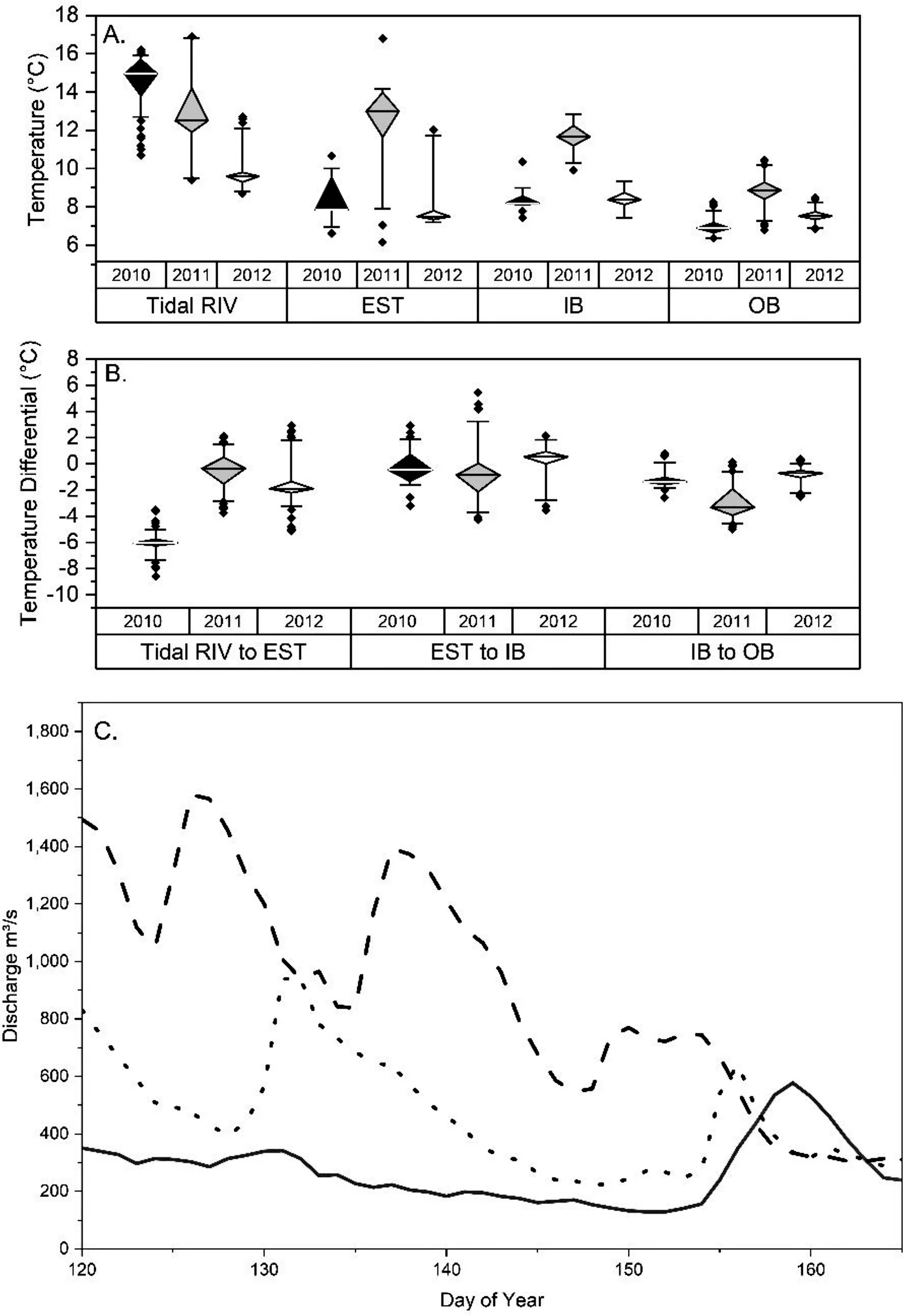
A and B – Temperature experience of all successful fish (both hatchery and natural smolts) transitioning across environments. Temperature data were paired with detections of individual smolts as they arrived at each of these environments. **A** – Temperature experience (°C) for smolts and postsmolts across environments by year. **B** – Temperature differentials (°C) between environments for hatchery and natural smolts and postsmolts by year. Diamonds = Outliers, Whiskers = 5th – 95th %, Box = 25th – 75th %, and “—“ = median value. Tidal RIV = Tidal River, EST = Estuary, IB = Middle Bay, OB = Outer Bay. **C** - Discharge (m³/s) for each of the three years. Black line = 2010, Dashed line = 2011 and Dotted line = 2012

Smolts and postsmolts encountered cooler temperatures as they traveled seaward (Fig. 2A). Tidal river temperatures ranged from 8.7 °C to 16.9 °C (median: 12.4 °C), estuarine temperatures from 6.2 °C to 14.2 °C (median: 9.5 °C), inner bay temperatures from 7.4 °C to 12.8 °C (median: 8.6 °C), and outer bay temperatures from 6.4 °C to 10.4 °C (median: 7.5 °C). All temperature differences were significant from one habitat to the next in each of the three years (all *P* < 0.05). The largest difference was observed in 2010, when the tidal river was 6.1 °C warmer than the estuary (Fig. 2B).

Discharge during the study period across the three years were different. In 2010, discharge exhibited the lowest and most stable baseline flow, hovering under 400 m³/s for much of the study period with a peak late (600 m³/s - Day 159; Fig. 2C). 2011 exhibited the highest overall volume and volatility, featuring two prominent peaks (1,600 m³/s - Day 126 and 1,400 m³/s - Day 137) which were the highest for the three years (Fig. 2C). In contrast, 2012 demonstrated an intermediate discharge pattern characterized by a single prominent peak of approximately 1,000 m³/s (Day 131), followed by a steady recession that plateaus between 200 and 400 m³/s (after Day 145; Fig. 2C).

### Movement rate and duration

In the tidal river, movement rates did not differ significantly between the origin groups in 2010 and 2011. However, in 2012, natural smolts (2.6 km/h) were significantly faster than hatchery smolts (1.6 km/h; W = 10.2, *P* < 0.001; Table 3). Movement rates increased from the tidal river to the estuary, with no significant difference between the origin groups (*P* > 0.05; Table 3). Within the bay, postsmolt movement rates for both origins decreased, with the only significant difference found in 2012, where NR smolts were faster (W = 5.7, *P* = 0.02; Table 3).

**Table 3:** Migration Movement Rates in kilometers per hour (km/h), of smolts and postsmolts across environments within the receiver network

Year	Median Discharge m^3^/s	Tidal River HTC/NR	C.I. Tidal River HTC/NR	TS/*P* value	Estuary HTC/NR	C.I. Estuary HTC/NR	TS/*P* value	Bay HTC/NR	C.I. Bay HTC/NR	TS/*P* value
2010	227.7 m^3^/s	2.2/2.5	0.9–3.2/ 1.2–3.5	1.9/0.17	3.3/2.9	2.0–4.3/2.2–4.0	0.3/0.56	1.3/1.2	0.9–2.3/ 0.8–2.1	1.6/0.21
2011	1,112.9 m^3^/s	2.1/2.0	0.9–3.1/ 1.3–3.6	0.2/0.69	4.6/3.8	3.1–5.9/3.2–4.7	2.1/0.14	1.6/2.0	0.9–2.6/0.6–2.8	0.3/0.61
2012	512.5 m^3^/s	**1.6/2.6**	**0.9–3.1/1.5–3.2**	**10.2/0.001**	4.6/4.2	3.2–6.2/3.0–5.8	1.0/0.31	**2.0/1.7**	**1.4–3.0/1.1–2.7**	**5.7/0.02**
Median Overall		**2.0/2.5**	**0.8–3.1/1.4–3.4**	**11.9/ < 0.001**	**4.2/3.8**	**2.7–5.7/ 2.5–4.8**	**7.8/0.005**	1.7/1.6	1.1–2.6/ 0.9–2.5	3.4/ 0.07

HTC = Hatchery; NR = Natural

No significant differences in duration throughout the entire array were detected between the origin groups in 2010 (hatchery = 3.8 d; natural = 3.7 d; W = 670.0, *P* = 0.62) or 2011 (hatchery = 3.8 d; natural = 4.0 d; W = 368.0, *P* = 0.90). In 2012, hatchery smolts (5.8 d) took significantly longer to emigrate than did natural smolts (4.2 d; W = 1380.0, *P* = 0.05). Overall, the median emigration duration was 4.2 d for hatchery smolts and 4.0 d for natural smolts, with no significant difference between the groups (W = 8284, *P* = 0.25).

### Path choice and distribution

**Fig. 3:**
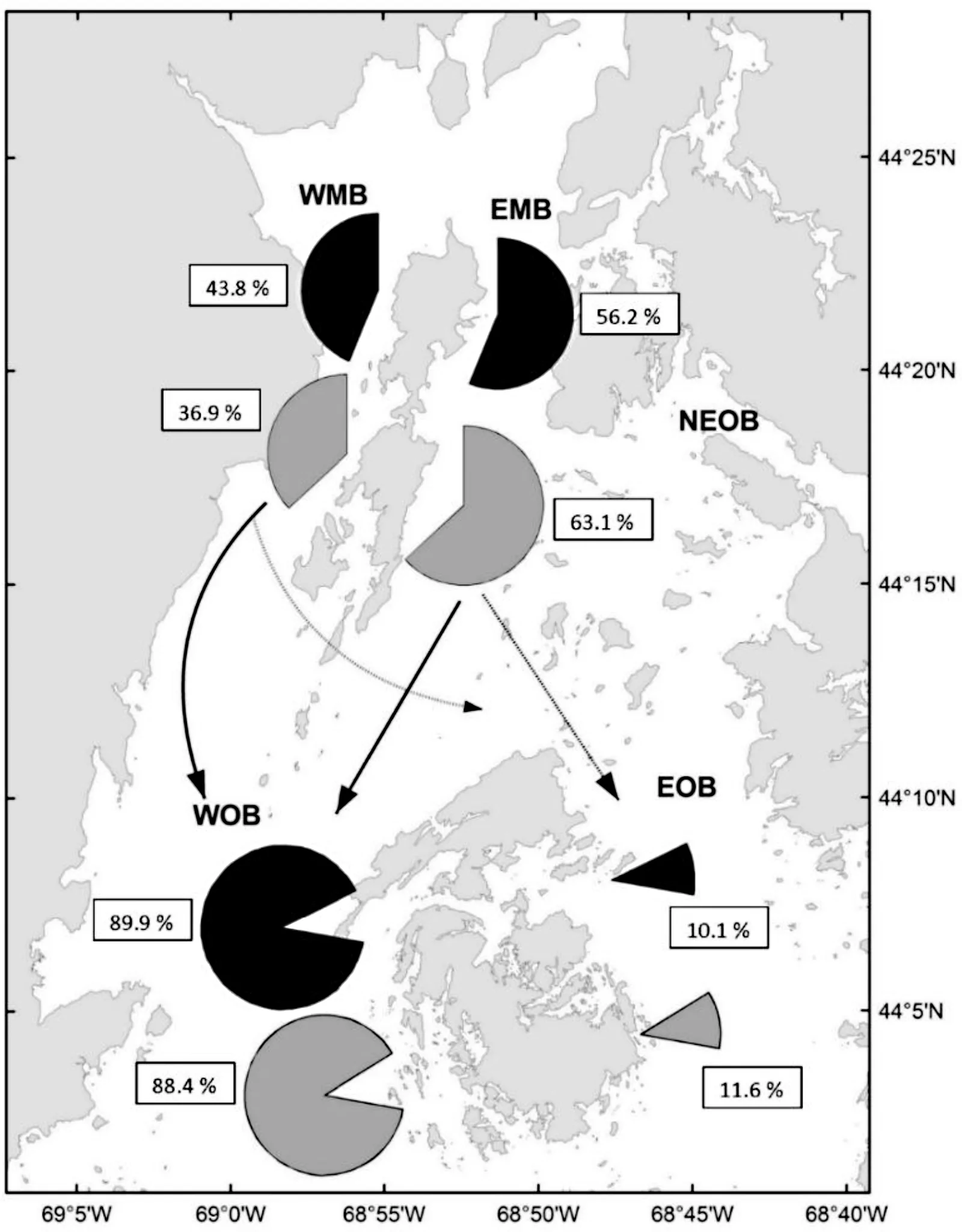
Cumulative path choice for hatchery (grey) and natural (black) postsmolts. Proportion of use is indicated by the pie-chart and corresponding percentages. Array locations: WMB – Western Middle Bay; EMB – Eastern Middle Bay; NEOB – Northeastern Outer Bay; WOB – Western Outer Bay; EOB – Eastern Outer Bay

In the middle bay, more than half of each origin group (149/236 = 63.1% hatchery; 59/105 = 56.2% natural) used EMB, and nearly 90% (183/207 = 88.4% hatchery; 80/89 = 89.9% natural) of each origin group traveled through the WOB (Fig. 3). No postsmolts were detected at NEOB during this study.

**Fig. 4:**
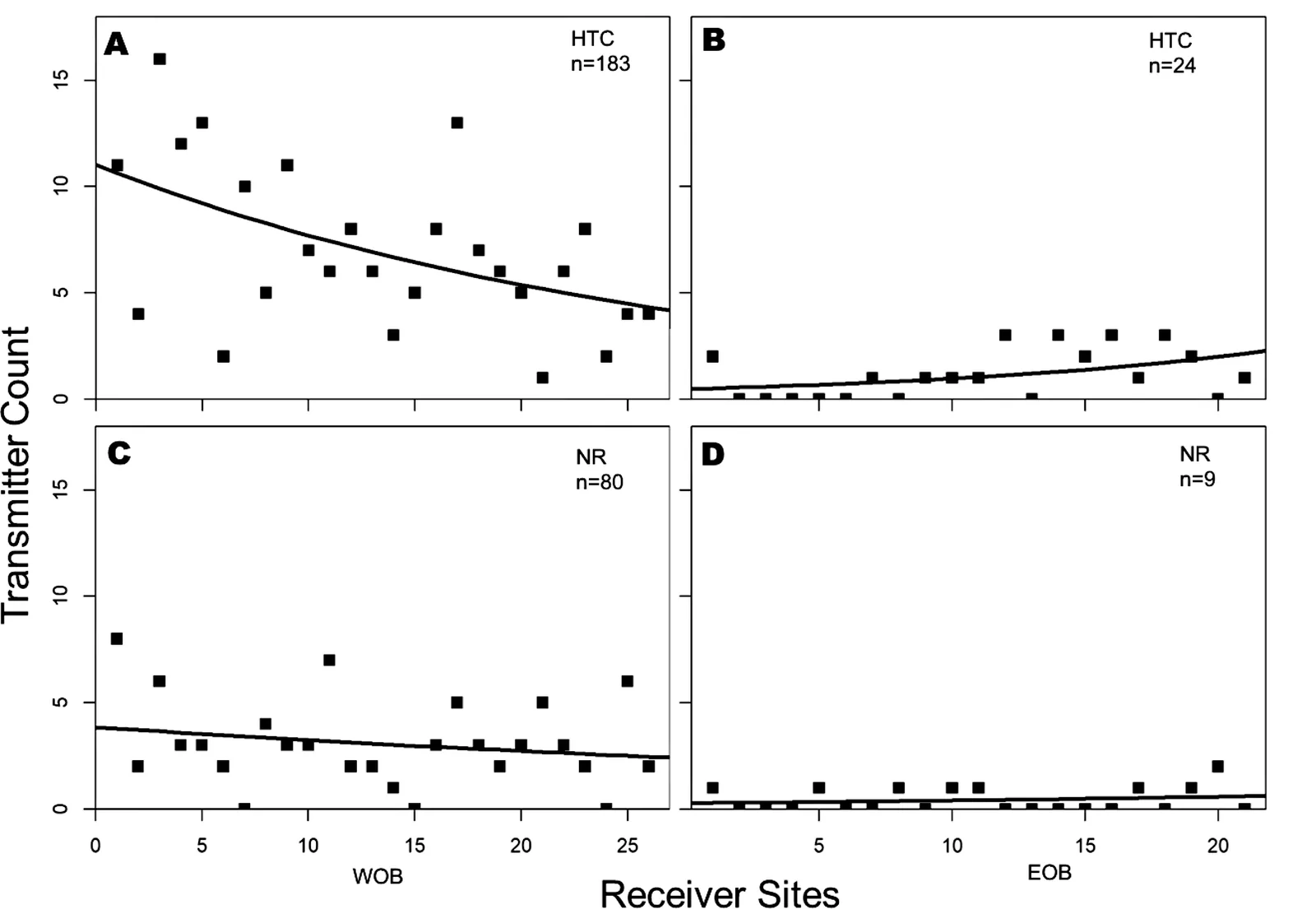
Distribution of postsmolts across the outer bay array prior to entry into the Gulf of Maine. Panel **A** – Hatchery (HTC) postsmolts along the Western Outer Bay (r^2^. = 0.248; z value =-3.57; *P* < 0.01); Panel **B** – Hatchery postsmolts along Eastern Outer Bay (EOB; r^2^= 0.182; z value = 0.36; *P* = 0.04); Panel **C** – Natural (NR) postsmolts along the WOB (r^2^ = 0.038; z value = -1.146; *P* = 0.25); Panel **D** – Natural postsmolts along the EOB (r^2^ = 0.026; z value = 0.66; *P* = 0.51). Receiver sites (1 - #) provide east to west orientation for each panel of the figure, n = the sample size of postsmolts represented within each of the panels

On the outer bay array, the postsmolt detection distribution was broad. In the WOB array, significantly more hatchery postsmolts were detected on the western side of the curtain array (r^2^ = 0.248; z value =-3.57; *P* < 0.01), whereas the natural postsmolts presented a similar, although not significant, pattern (r^2^ = 0.038; z value = -1.146; *P* = 0.25; Fig. 4 panels A and C). On the EOB array, hatchery postsmolts (*n* = 24) were detected in significant numbers on the eastern side of the curtain array (r^2^= 0.182; z value = 0.36; *P* = 0.04), whereas natural postsmolt detections (*n* = 9) were not significant and exhibited no pattern (r^2^ = 0.026; z value = 0.66; *P* = 0.51; Fig. 4 panels B and D).

### Survival

Following the first phase of model selection, in which survival was held constant and detection probability (p) was allowed to vary, the top-ranked model included differences in detection probability between reaches and years: p (reach + year) (Table S.1). While one other model p (reach + year + origin) had a $\:{{\Delta\:}}_{AICc}<2$, this model increased the complexity and number of parameters, so, we used the best and most parsimonious model. The detection probabilities (p) within the entire study area were high and ranged from 0.83 to 0.99 in the tidal river, 0.80–0.99 in the estuary, and 0.89–1.0 in the bay.

**Fig. 5:**
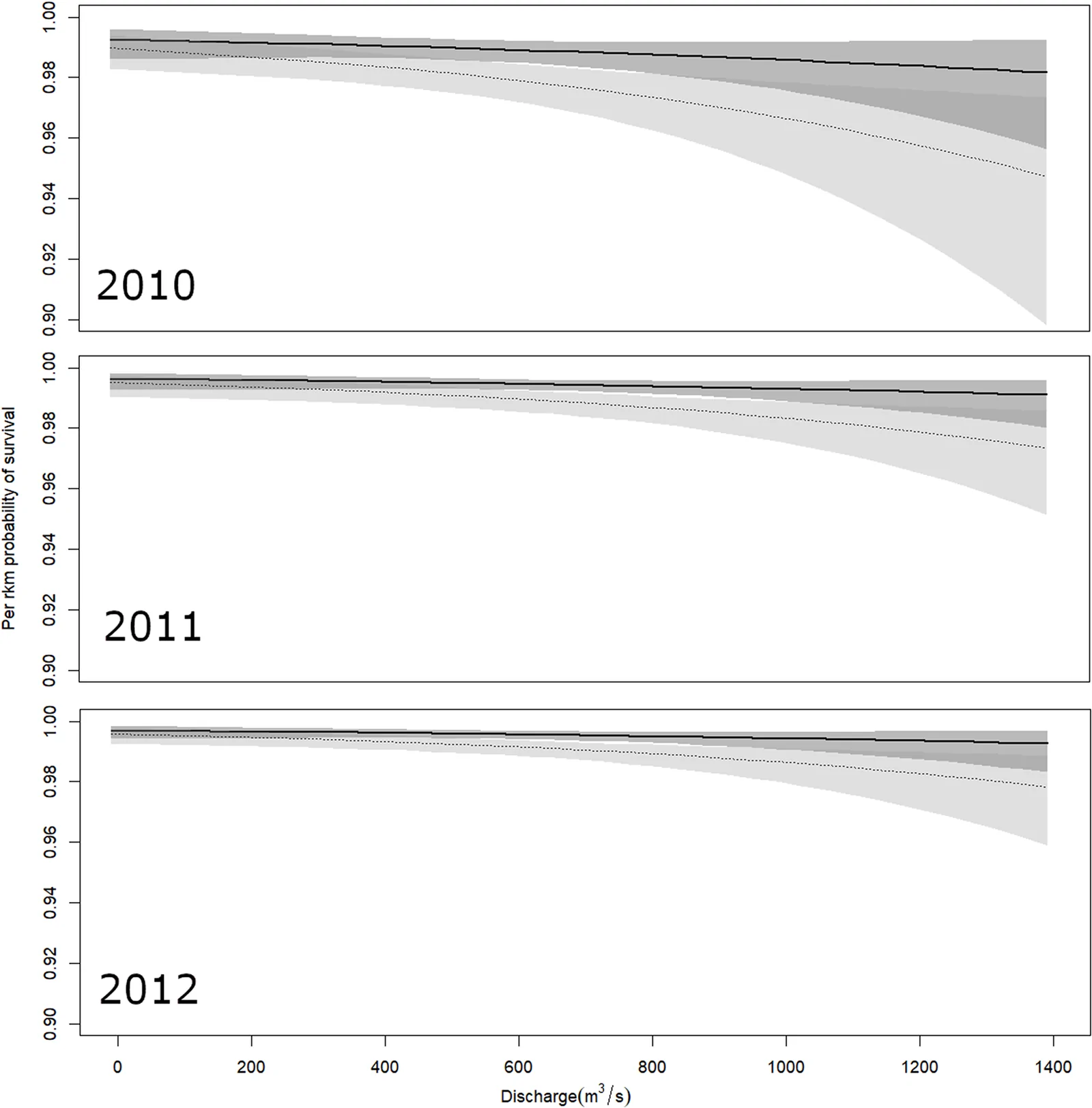
Effect of discharge (m^3^/s) on apparent survival across years. Dark gray with solid line = hatchery and light gray with dotted line = natural smolts

**Fig. 6:**
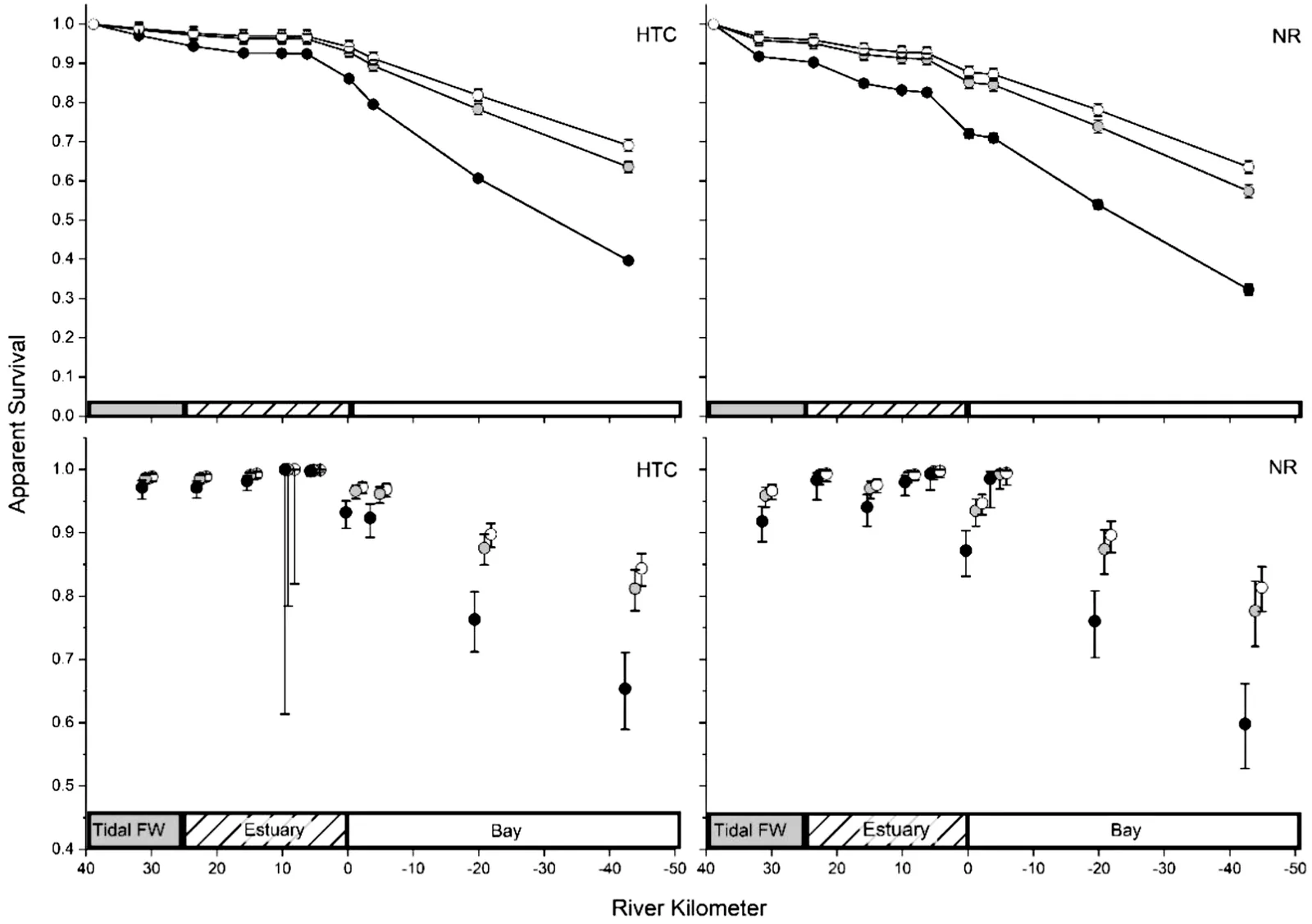
Apparent survival of smolts between groups and years. Top Panel - Cumulative and Bottom Panel - Per kilometer survival. Hatchery (HTC) and natural (NR) smolts. Black = 2010, Gray = 2011, and White = 2012

The best fitting model was $\:{\Phi\:}\:({\sim}reach\times\:origin\times\:discharge\times\:year$) p (~ reach + year). This represents an interactive effect of reach, origin, discharge and year (i.e., each estimate was different). The next best model had a ΔAIC > 35 (Table 4; Table S.2), resulting in overwhelming support for the first model. We explored the effects of all the explanatory variables from the first model. Survival differences between reaches are observable in Fig. 6 (bottom panels), with per-rkm survival ranging from 0.53 to 1, and yearly survival being highest in 2012, followed by 2011, and lowest in 2010. Discharge had a negative effect on survival (Fig. 5), increasing the mortality of natural smolts. The negative discharge effect should be interpreted conditionally within the modeled release/migration period, not as a simple among-year comparison of annual cumulative survival. In our analysis, discharge was assigned at the fish/day level and modeled together with year (and, in some candidate sets, with reach and origin interactions). Thus, although annual cumulative survival in Fig. 6 was lower in 2010, the fitted covariate relationship indicates that within a given year/reach context, higher discharge was associated with lower survival.

**Table 4:** Model selection statistics from phase 2 of model selection for Atlantic salmon smolt survival in the Penobscot River, 2010–2012 (Φ = survival; p = detection probability)

Model	k	AICc	ΔAICi	W
Φ(~ time * origin * dis * year) P (~ time + year)	117	13074.2	0.00	1
Φ(~ time * origin * ss * year) P (~ time + year)	93	13109.87	35.67	0
Φ(~ time * origin * dis + I(dis^2) * year) P (~ time + year)	56	13333.55	259.35	0
Φ(~ time * origin * diff * year) P (~ time + year)	129	13355.66	281.45	0
Φ(~ time * dis + year + origin) P (~ time + year)	32	13375.21	301.01	0

Variables represented in candidate models were, dis (discharge), diff (temp differential within the estuary), ss (smolt score), and origin. The number of parameters (k), Akaike’s information criterion corrected for small sample size (AICc), the difference in AICc between the best model and the ith model (Δi), and relative probability of the ith model being the best model (Akaike weight; wi) are provided. Presented are top five models, with all models presented in Supplementary Table S.2

The cumulative survival of hatchery smolts (range: 0.40–0.69) was greater than that of natural smolts (range: 0.32–0.64) each year in this study (Fig. 6, Top Panel). Although hatchery smolts presented greater cumulative survival, the patterns of loss were similar for both origins (Fig. 6, Top Panel).

Hatchery smolts had similar or greater survival per-rkm than did natural smolts within most intervals and environments (Fig. 6, bottom panel). In the tidal river, hatchery smolts exhibited moderate-to-high survival (0.97–0.99), whereas natural smolts presented low-to-moderate survival (0.92–0.97; Fig. 6, bottom panels). Within the estuary, survival for both origin groups were broad, with hatchery smolts observed at 0.93–0.99 and natural smolts at 0.87–0.99. The lowest estuary survival rates were found just prior to the inner bay (0.87–0.97; Fig. 6, bottom panel). Postsmolt survival for both origins were moderate-to-low during bay entry and decreased with seaward progression; inner (hatchery = 0.92–0.96; natural = 0.98–0.99), middle (hatchery = 0.76–0.90; natural = 0.76–0.89) and outer bay (hatchery = 0.65–0.84; natural = 0.60–0.81; Fig. 6, bottom panel) for each origin. Apparent survival differed significantly among origins. The model including origin in the survival component fit the data better than the otherwise identical reduced model excluding origin, based on a likelihood ratio test, χ² = 478.56, df = 81, *p* < 0.00001. This result indicates that origin explained significant variation in apparent survival after accounting for time, year, and discharge.

Compared with Thorstad et al. [62], mortality in tidal freshwater was comparable (0.02 in Thorstad et al., vs. 0.01 for hatchery smolts and 0.03 for natural smolts in the present study). However, the estuarine mortality reported by Thorstad et al., (0.06) was at least three times greater than that reported in this study (0.01 for hatchery smolts and 0.02 for natural smolts). Conversely, mortality within the bay was more than 10 times greater in this study (0.12 for hatchery smolts and 0.13 for natural smolts) than in Thorstad et al., (0.01; Figure S.1).

## Discussion

Using a three-year acoustic tagging dataset, we tracked the movement of smolts from two origins—hatchery and natural—from the Penobscot River through the estuary and into the Gulf of Maine. Although other investigations have compared the performance of hatchery and natural fish, most have focused on dam passage and in-river assessments [24, 60], with limited investigations in estuary and marine environments. This is the first effort to describe the timing, duration, path choice, and survival of a paired release in this system. Hatchery smolts were significantly larger than their natural counterparts and, in most years, showed no significant difference in movement rate, migration duration, or path choice. Notably, hatchery smolts survived at a higher rate than natural smolts across these three years of paired releases within a portion of the spring migration period.

Independent of origin, mortality was consistent with the findings of local and regional studies [20, 21, 23, 33, 37, 53, 59]. However, notable differences were observed compared with broader syntheses of Atlantic salmon smolt dynamics, such as Thorstad et al. [62]; (Figure S.1), who summarized findings from North American and European rivers. While smolt mortality reported by Thorstad et al. [62] was comparable to our findings (per km) within tidal freshwater, their observed mortality was greater in the estuary and significantly lower in the bay. Differences in mortality rates are expected due to the broad geographical distinctions among these studies, as well as variations in habitat, study design, and predator assemblages across the species range. The specific factors driving these mortality differences remain poorly understood. However, the observation that regional populations (e.g., those in proximity to the Gulf of Maine) exhibit similar responses to habitat-specific losses suggests a common challenge.

While findings have shown notable differences compared with existing syntheses [62], they also contrast with many head-to-head evaluations of tagged hatchery versus wild (or natural) smolts (Atlantic and Pacific), indicating that natural origin salmon with longer riverine residences perform as well or better than their hatchery counterparts do [10, 26, 28, 38, 61, 65]. Factors such as husbandry, physical conditions and stocking strategies have been linked to lower survival of hatchery smolts [2, 41, 42]. In contrast to these findings, this study revealed that hatchery smolts presented higher cumulative and habitat-specific per-rkm survival rates than did natural smolts. Interactions at dams within this watershed have been deemed impactful, with immediate and latent results [59]. We hypothesize that the interaction with the Weldon dam bypass during natural smolt collection was a significant contributor to these differences in cumulative survival. These findings are counter to other studies of river-reared fish (previously noted), as well as what smolt-to-adult return rates, which are consistently higher than hatchery stocked smolts within in the Gulf of Maine [67].

Multiple factors impacting saltwater transition and survival also warrant consideration for these two origin groups. The hatchery smolts in this study were significantly larger than the natural smolts (median FL: 195.0 mm vs. 172.0 mm; Table 1), which is consistent between river-reared fish and restoration smolt stockings in Maine [16, 66, 67]. Although smolt size has been linked with migration success in other studies [9, 31, 55], it was not a significant covariate in the best-fitting model. By exceeding the threshold of published criteria [36] for smolt selection and confirming the model results, we are confident that there were no size-based effects. Models incorporating rearing origin consistently demonstrated a better fit than those using fork length (FL) as an explanatory variable, suggesting that rearing origin is a stronger predictor of survival.

Beyond physical characteristics, our results indicate that physiological readiness—measured via smolt score—differed between origin groups. Hatchery smolts exhibited higher mean smolt scores, suggesting greater physiological preparedness. This is notable, as Stich et al., [59] demonstrated that higher physiological readiness correlates with increased probability of successful estuary passage. However, hatchery smolts from the accelerated Gulf of Maine restoration program undergo distinct rearing experiences compared to natural smolts, which may drive observed behavioral differences, such as out-of-sync migration in the Penobscot River [16] and condensed run times in neighboring smolt stocked rivers [15]. These behaviors may stem from rearing history [59, 74], specifically differing thermal history in the months leading up to emigration, which may influence physiological development and stress responses, warranting further investigation into their impacts on early marine survival. Alternatively, underlying genetic factors may contribute to poor marine survival [34, 42] and also require consideration. While dam interaction effects remain relevant, these additional physiological and genetic factors influencing saltwater transition justify further study.

In addition to origin, discharge exhibited the greatest interannual variability and was a key covariate in the best-ranking model. Although release-day discharge was strongly associated with survival, fish experienced no more than five distinct discharge values within each year, and discharge was partially confounded with release date. Thus, the observed relationship may partly reflect differences among release cohorts and should not be interpreted as causal. Nevertheless, because discharge did not vary monotonically with release date, the relationship suggests that relative flow conditions could help inform release timing, pending validation across additional release dates and year. For both origins, we found that survival decreased with increasing discharge (Fig. 5). We hypothesize that the exposure of natural smolts to debris and complex bypass conditions contributed to this pattern; however, the impact—albeit slight—on hatchery smolt survival remains unclear. High discharge also has the potential to impact detection efficiency in some studies [14], which ultimately could bias survival estimates. To address concerns about detection efficiency bias within this study area, Goulette and Hawkes, [18] conducted an assessment using transmitter-tagged smolts and fixed pulse transmitters during two of the three study years (including the high discharge year, 2011). They determined that discharge alone did not significantly impact the detection efficiency. While the interaction of discharge and flood tides did reduce efficiency, this is considered of little concern because of the consistent nature of smolt movements observed across origin groups and study years. This study of tagged smolts revealed high overall efficiencies ranging from 0.80 (lowest estimated detection rate in the estuary) to 1.0 in the bay.

Mortality notably increased at rkm 0.0 (river mouth), an area characterized by consistent salinities of at least 20 ppt and significant temperature gradients. This observation of heightened mortality at the river mouth aligns with findings from Hawkes et al., [23] in the nearby Dennys River, where smolts experienced rapid inundation with cold, high-salinity waters from the Bay of Fundy, coinciding with initial postsmolt losses. The relative contributions of salinity, low temperature, temperature differentials, or their combination [1, 25, 30] and/or the significant presence of known predators [39, 44] to these observed mortalities remain unclear. It is not known whether these observed gradients always existed in the spring or if changes have occurred within this system. Regardless, the advancing phenology of Atlantic salmon smolt run-time throughout their range [32, 51] highlights the need for further study of these environments and ecosystems, particularly given the high sensitivity of Atlantic salmon to environmental change [22] and how these shifts might alter exposure to the observed gradients.

In addition to salinity and temperature, the movement rates of the emigrating smolts also provided insights into their migration in the bay. Through the river and estuary, movement rates were much higher, with smolts using river discharge during passive transport. Within the bay, movement slowed with seaward progression, which is consistent with smolt and postsmolt movements within this system and within the range of Atlantic salmon [53, 59, 62]. The majority of postsmolts use the eastern side of the middle bay, where wind direction may define changes in outflow, but is generally found to the east of Islesboro [73]. Once through the middle bay, egress of most postsmolts into the Gulf of Maine occurred through the western outer bay. The westerly orientation of these outgoing postsmolts is consistent with survey [56] and telemetry [53] data collected within the bay. The information we obtained on smolt use of migration corridors will enable managers to make informed decisions regarding the development of infrastructure and renewable resource projects within Penobscot Bay [56].

Given these findings within a broader conservation context, hatcheries serve distinct purposes for Atlantic and Pacific salmon populations, reflecting their varying conservation statuses and management goals. Remnant populations of Atlantic salmon are protected under the ESA, whereas Pacific salmon exhibit a broad range of abundance, from ESA-listed species to those supporting records of commercial catches [5, 6]. Hatcheries are employed on both coasts; Pacific salmon supplement recreational and commercial fisheries [54], and both Atlantic salmon and Pacific salmon are utilized in population restoration efforts [67, 71]. While hatcheries carry considerable risk, including impacts on carrying capacity, competition, interbreeding, and displacement, as well as genetic impacts; [6, 43], they remain a necessary tool for population recovery [40, 71].

## Conclusions

For the GOM DPS, the conservation hatchery stocking program remains the difference between persistence and extirpation [47, 67]. Given that the GOM DPS represents the last remaining population of Atlantic salmon in the United States, continuing restoration stocking is critical to prevent continued range constriction at the extant southern limit. Rebuilding the GOM DPS of Atlantic salmon requires that more smolts survive to reach the ocean and successfully return to rivers as reproductive adults. Managers need to balance their approach in two phases: (1) increasing the number of Atlantic salmon reaching the ocean through hatchery supplementation and (2) continuing to improve the passage and mitigation measures for increasing the survival of natural smolts. Improvements in passage will justify reseeding vacant habitats in the upper reaches of the Penobscot River, which has the ability to produce approximately one million additional natural smolts [57]. In the meantime, managers must work to improve smolt survival during emigration and continue to utilize hatchery smolts to provide a demographic advantage to increase smolt numbers and adult returns. Implementing the best available tools and diverse spatial and temporal stocking strategies (e.g., [15, 16]) may optimize smolt estuarine entry timing and improve survival, yielding the necessary demographic boost.

## Supplementary Information

Below is the link to the electronic supplementary material.

Supplementary Material 1

## Data Availability

The datasets generated and/or analysed during the current study are available in the Northeast Fisheries Science Center Atlantic salmon Telemetry Monitoring Repository, https://www.fisheries.noaa.gov/inport/item/27492.
