# Supplementary material for: Migration dynamics of acoustic-tagged hatchery and natural atlantic salmon smolts
Source: BMC Zool. 2026 Jul 23;11:34. doi: 10.1186/s40850-026-00278-y (PMC13682133; doi:10.1186/s40850-026-00278-y)
Supplement: Supplementary file 1 [file 40850_2026_278_MOESM1_ESM.docx]

Supplementary Table S.1. Model selection statistics from phase 1 of model selection for Atlantic Salmon smolt survival in the Penobsoct River, 2010 - 2012 (p = detection probability). Survival (Φ) was held constant (.) for detection model selection, and variables used in candidate detection models included origin, reach (t) and year. The number of parameters (k), Akaike’s information criterion corrected for small sample size (AICc), the difference in AICc between the best model and the ith model (Δi), and relative probability of the ith model being the best model Akaike weight; wi) are provided. All models are shown.

| **Model** | **k** | **AICc** | **Δi** | **wi** |
| --- | --- | --- | --- | --- |
| Φ_(.)_*P*_(t + year)_ | 12 | 13943.13 | 0 | 0.5 |
| Φ_(.)_*P*_(t + year + origin)_ | 13 | 13943.14 | 0.01 | 0.49 |
| Φ_(.)_*P*_(t * origin)_ | 19 | 13987.26 | 44.13 | <0.01 |
| Φ_(.)_*P*_(t )_ | 10 | 14010.74 | 67.61 | <0.01 |
| Φ_(.)_*P*_(t + origin)_ | 11 | 14012.22 | 69.09 | <0.01 |
| Φ_(.)_*P*_(year * origin)_ | 7 | 14060.76 | 117.64 | <0.01 |
| Φ_(.)_*P*_(year + origin)_ | 5 | 14072.02 | 128.89 | <0.01 |
| Φ_(.)_*P*_(year)_ | 4 | 14072.17 | 129.04 | <0.01 |
| Φ_(.)_*P*_(origin)_ | 3 | 14140.01 | 196.89 | <0.01 |
| Φ_(.)_*P*_(t * year)_ | 28 | 15900.57 | 1957.44 | <0.01 |

Supplementary Table S.2. Model selection statistics from phase 2 of model selection for

Atlantic Salmon smolt survival in the Penobscot River, 2010 – 2012 (Φ= survival; p = detection

probability). Variables represented in candidate models were dis (discharge), diff (temp differential within the estuary), ss (smolt score), and origin. The number of parameters (k), Akaike’s information criterion corrected for small sample size (AICc), the difference in AICc between the best model and the ith model (Δi), and relative probability of the ith model being the best model (Akaike weight; wi) are provided. All models are shown.

| **model** | **k** | **AICc** | **ΔAICi** | **W** |
| --- | --- | --- | --- | --- |
| Φ(~time * origin * dis * year) P (~time + year) | 117 | 13074.2 | 0.00 | 1 |
| Φ(~time * origin * ss * year) P (~time + year) | 93 | 13109.87 | 35.67 | 0 |
| Φ(~time * origin * dis + I(dis^2) * year) P (~time + year) | 56 | 13333.55 | 259.35 | 0 |
| Φ(~time * origin * diff * year) P (~time + year) | 129 | 13355.66 | 281.45 | 0 |
| Φ(~time * dis + year + origin) P (~time + year) | 32 | 13375.21 | 301.01 | 0 |
| Φ(~time * dis + I(dis^2) + year + origin) P (~time + year) | 32 | 13375.21 | 301.01 | 0 |
| Φ(~time + origin * dis + year) P (~time + year) | 25 | 13390.02 | 315.82 | 0 |
| Φ(~time + origin + year + dis) P (~time + year) | 24 | 13390.45 | 316.25 | 0 |
| Φ(~time * origin * dis) P (~time + year) | 51 | 13392.15 | 317.95 | 0 |
| Φ(~time * origin + year) P (~time + year) | 31 | 13392.52 | 318.32 | 0 |
| Φ(~time + dis + year) P (~time + year) | 23 | 13394.61 | 320.40 | 0 |
| Φ(~time + origin * diff + year) P (~time + year) | 25 | 13403.87 | 329.67 | 0 |
| Φ(~time * ss + year + origin) P (~time + year) | 32 | 13411.55 | 337.35 | 0 |
| Φ(~time * origin * diff) P (~time + year) | 47 | 13416.01 | 341.81 | 0 |
| Φ(~time + origin * ss + year) P (~time + year) | 25 | 13420.26 | 346.05 | 0 |
| Φ(~time + origin + year + diff) P (~time + year) | 24 | 13421.55 | 347.35 | 0 |
| Φ(~time + origin + year) P (~time + year) | 23 | 13426.33 | 352.13 | 0 |
| Φ(~time + origin + year + ss) P (~time + year) | 24 | 13427.64 | 353.44 | 0 |
| Φ(~time + year + ss) P (~time + year) | 23 | 13433.22 | 359.02 | 0 |
| Φ(~time + year + ss) P (~time + year) | 23 | 13433.22 | 359.02 | 0 |
| Φ(~time * origin * ss) P (~time + year) | 47 | 13438.54 | 364.33 | 0 |
| Φ(~time * origin) P (~time + year) | 29 | 13454.35 | 380.14 | 0 |
| Φ(~time * dis + origin) P (~time + year) | 30 | 13460.89 | 386.69 | 0 |
| Φ(~time * dis + I(dis^2) + origin) P (~time + year) | 30 | 13460.89 | 386.69 | 0 |
| Φ(~time * ss + origin) P (~time + year) | 30 | 13470.59 | 396.39 | 0 |
| Φ(~time + origin) P (~time + year) | 21 | 13488.23 | 414.03 | 0 |
| Φ(~time * diff + origin) P (~time + year) | 30 | 13494.36 | 420.16 | 0 |
| Φ(~time + dis) P (~time + year) | 21 | 13504.66 | 430.45 | 0 |
| Φ(~time + ss) P (~time + year) | 21 | 13504.77 | 430.57 | 0 |
| Φ(~origin + year + dis) P (~time + year) | 16 | 13851.63 | 777.43 | 0 |
| Φ(~1) P (~time + year) | 12 | 13943.13 | 868.92 | 0 |

Supplement Figure S.1. Standardized proportion of smolt mortality (km^-1^) across environments in relation to Thorstad et al.’s Figure 1 (2012). Black box represents our dat covering 2010 to 2012, the box represents the 25^th^ and 75^th^ percentile, black line within box represents the median value, and whiskers represent min and max. Thorstad et al. (2012) median plot includes median (circle) along with minimum and maximum value for whiskers. HTC = Hatchery Reared Smolts, NR = Naturally Reared Smolts.
